# Differential representation of feedback and decision in adolescents and adults

**DOI:** 10.1016/j.neuropsychologia.2014.01.021

**Published:** 2014-04

**Authors:** Amir Homayoun Javadi, Dirk H.K. Schmidt, Michael N. Smolka

**Affiliations:** a Department of Psychiatry and Neuroimaging Center, Technische Universität Dresden, Dresden, Germany; b Institute of Behavioural Neuroscience, Department of Cognitive, Perceptual and Brain Sciences, University College London, London, UK

**Keywords:** Developmental, Uncertainty, Anterior cingulate cortex (ACC), Ventral striatum (VS), Ventromedial prefrontal cortex (vmPFC), Reward processing, Decision-making, Probabilistic reversal learning

## Abstract

It is widely accepted that brain maturation from adolescence to adulthood contributes to substantial behavioural changes. Despite this, however, knowledge of the precise mechanisms is still sparse. We used fMRI to investigate developmental differences between healthy adolescents (age range 14–15) and adults (age range 20–39) in feedback-related decision making using a probabilistic reversal learning task. Conventionally groups are compared based on continuous values of blood oxygen level dependent (BOLD) percentage signal change. In contrast, we transformed these values into discrete states and used the pattern of these states to compare groups. We focused our analysis on anterior cingulate cortex (ACC), ventral striatum (VS) and ventromedial prefrontal cortex (vmPFC) as their functions have been shown to be critical in feedback related decision making. Discretisation of continuous BOLD values revealed differential patterns of activity as compared to conventional statistical methods. Results showed differential representation of feedback and decision in ACC and vmPFC between adolescents and adults but no difference in VS. We argue that the pattern of activity of ACC, vmPFC and VS in adolescents resulted in several drawbacks in decision making such as redundant and imprecise representation of decision and subsequently poorer performance in terms of the number of system changes (change of contingencies). This method can be effectively used to infer group differences from within-group analysis rather than studying the differences by direct between-group comparisons.

## Highlights

• ACC activity in adults represented solely the subsequent decision.
• ACC activity in adolescents reflected both feedback and decision.
• Activity of vmPFC in adults reflected both feedback and decision.
• Activity of vmPFC in adolescents it represented feedback only.
• VS represented feedback and did not differ between adolescents and adults.

## 1 Introduction

When making decisions, adolescents generally show a propensity towards risk-taking and novelty-seeking due to the greater lure of positive consequences, as well as the considerable influence of social context, resulting in the disregard of disregarding negative consequences (Crews, He, & Hodge, 2007; Dahl, 2004; Steinberg, 1987). For example, self-report and observational studies have shown that adolescents are involved in the majority of traffic accidents (Furby & Beyth-Marom, 1992; Steinberg, 2004). These individuals also have a higher chance of engaging in criminal behaviour, substance abuse and unsafe sexual activity. Such behaviour has been associated with an “imbalance” in the development of different brain areas in adolescents (Cohen et al., 2010).

Maturation of the human brain and reorganisation of neuronal structures related to emotional, motivational and cognitive processes are essential for the establishment of behavioural control, cognitive flexibility and efficient brain function. Differences in the pattern of development of reward and control-related circuitry have been proposed to lead to an “imbalance” in the adolescent brain, presumably due to immature frontal lobe suppression of reward sensitivity in mesolimbic regions (Casey, Jones, & Hare, 2008; Gogtay et al., 2004). Behavioural changes in risk-taking observable during development might be explained by an imbalance between early maturing mesolimbic brain regions, namely the ventral striatum functionally associated with affective information processing, relative to less mature prefrontal areas, critically involved in top-down control (Figner, Mackinlay, Wilkening, & Weber, 2009; Somerville & Casey, 2010; Somerville, Jones, & Casey, 2010). As a result, compared to adults, adolescents place greater value on the potential positive (as opposed to negative) consequences of risk-taking (Ernst, Pine, & Hardin, 2006; Steinberg, 2010). Nevertheless the literature is inconsistent and therefore it is highly debated whether reward-related striatal brain activity is exaggerated or attenuated in the adolescent brain. Some neuroimaging studies found the striatum to be hypersensitive during reward processing (Cohen et al., 2010; Galvan et al., 2006; Geier, Terwilliger, Teslovich, Velanova, & Luna, 2010) while others report hyposensitivity on rewards in striatal regions (Bjork et al., 2004; Geier et al., 2010) in adolescents.

Albeit this inconsistency several studies have shown differences in terms of behavioural performance between adolescents and adults in a large variety of reward-related tasks. For instance adults perform better when choosing between high- and low-risk or during feedback-based learning (Cauffman et al., 2010; Galvan et al., 2006; van Leijenhorst et al., 2010). Using a deterministic reversal learning task van der Schaaf, Warmerdam, Crone, and Cools (2011) found that overall performance increases from age 10 to 25. Interestingly, punishment-based learning was best for the youngest age group, whereas reward-based learning was best in young adults.

These differences in behaviour and brain activity have increasingly attracted attention to developmental studies of the brain and behaviour of adolescents. In this context we aimed to investigate their differences in the context of feedback-related decision making.

We used a probabilistic reversal learning (PREL) task to investigate how adolescents and adults incorporate feedback (both rewarding and punishing) in their decisions in a dynamic and uncertain environment, where feedback is probabilistic and contingencies change from time to time. PREL has been used previously in many studies investigating feedback-related decision making using behavioural (Dickstein et al., 2010; Dombrovski et al., 2010), brain imaging (Budhani, Marsh, Pine, & Blair, 2007; Chase, Swainson, Durham, Benham, & Cools, 2011; Cools, Clark, Owen, & Robbins, 2002; O׳Doherty et al., 2004) and computational modelling (Hampton, Bossaerts, & O׳Doherty, 2006; Hampton & O׳Doherty, 2007; Krugel, Biele, Mohr, Li, & Heekeren, 2009) approaches.

Three main brain regions that have been implicated with probabilistic reversal learning are anterior cingulate cortex (ACC), ventral striatum (VS) and ventromedial prefrontal cortex (vmPFC). It has been shown that ACC is crucial for the processing of feedback-related decision-making and error management (Kennerley, Walton, Behrens, Buckley, & Rushworth, 2006), for a review see (Rushworth & Behrens, 2008). The difference between the expected value and the actual outcome of an action, known as reward prediction error (PE), is encoded in the VS (Cohen et al., 2010; Hampton et al., 2006; Krugel et al., 2009). vmPFC has been found to be critically important in *reversal* learning (for a review see (Clark, Cools, & Robbins, 2004). As such, we focused our analysis on these brain areas.

**Fig. 1:**
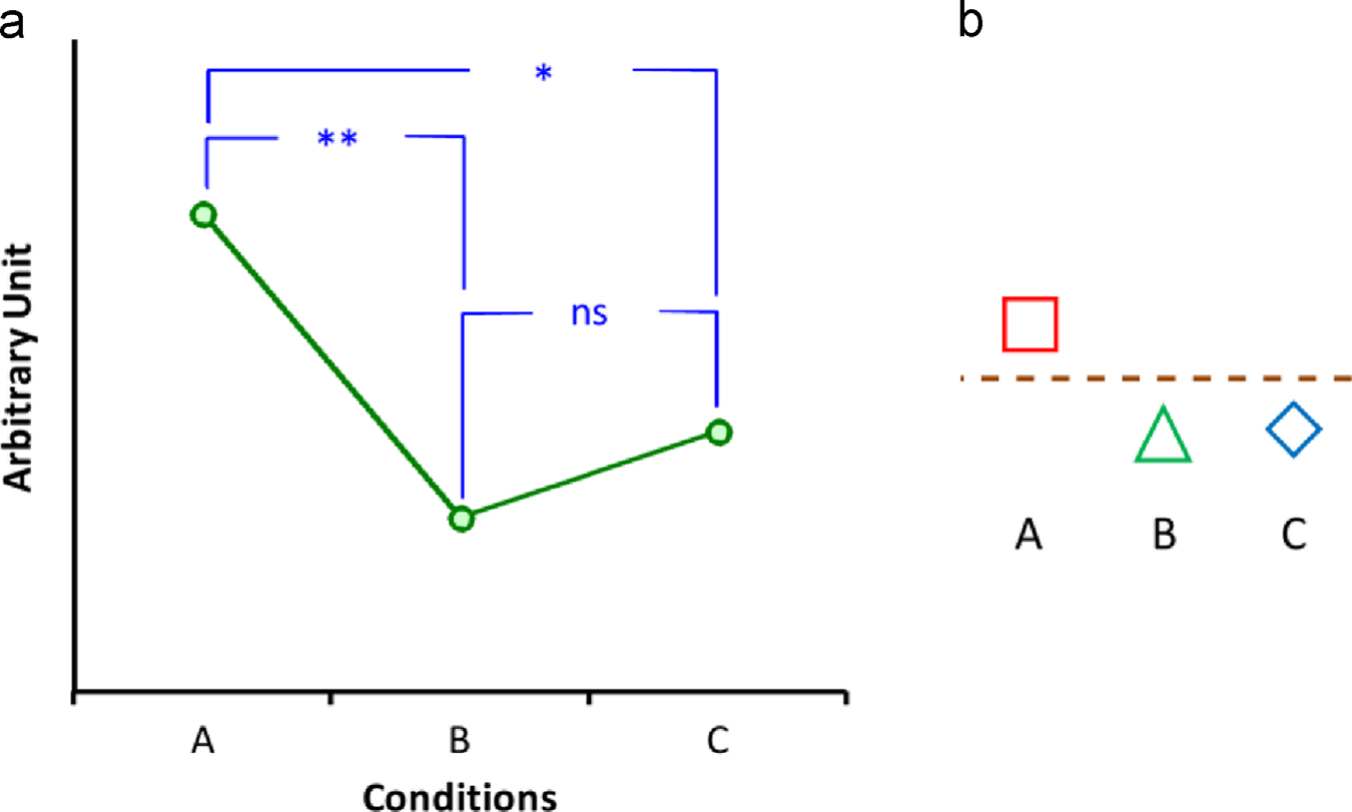
An example of discretisation of continuous data with three conditions of *A*, *B* and *C* into *states*. Vertical axis in (a) shows BOLD percentage signal change. Values in (a) in converted into states as shown in (b). (b) is constructed based on significant and non-significant differences shown in (a): condition *A* is significantly different from *B* and *C* solely based on significant difference and not the literal value. On the other hand, conditions *B* and *C* are classified into the same state as they are not significantly different, although they are numerically different. It should be emphasised that there is no order or level to states contrary to continuous values in which we have qualities like smaller and bigger, or before and after. State of condition *A* is placed above state of conditions *B* and *C* solely for the sake of easier association with quantitative data. ^⁎^ and ^⁎⁎^ show significant difference, ^ns^ non-significant difference.

To date, direct comparison of groups has provided us with a rich body of knowledge. We, however, were not interested in how adolescent and adult brain activity differed in a given condition, but in a more abstract and functional comparison between groups. Contrary to conventional methods of comparison in which groups are directly compared using between-group tests, we compared groups by converting brain activity into discrete states based on a 2-level randomisation procedure. Blood oxygen level dependent (BOLD) percentage signal changes in different conditions were transformed into states of activity, i.e. continuous percentage signal changes were converted into a few discrete states. This conversion enabled us to remove baseline differences and overcome intrinsic scale differences between adolescents and adults groups. Fig. 1 shows an example of how this conversion was carried out. These states represented different conditions in the brain in terms of feedback and decision for the subsequent trial. The patterns of these states in different brain areas were then compared between adolescents and adults. This method is comparable with drawing conclusions on differences between groups from within-group comparisons.

## 2 Methods

### 2.1 Participants

The data from adolescents were acquired as part of the project “The adolescent brain” funded by the German Federal Ministry of Education and Research (BMBF). This project aims to investigate structural and functional brain development in the context of environmental and genetic factors. The study has a longitudinal design and seeks to reveal links between functional as well as structural brain development and occurrence of substance use disorders. We present results of the first acquisition wave of adolescent data in a cross-sectional design compared to data of an adult group.

260 adolescents were recruited from local secondary schools (adolescent group). We had to exclude 40 adolescents from the analysis due to acute head movements (movements greater than 3 mm in any one direction), interruption in scanning, fault in data transfer or missing data. Consequently we analysed data for 220 adolescents (115 male (52.27%), age range 14–15, mean age 14.61 years (SD=0.32)). As a control group we recruited 28 adult participants (adult group) by board and Internet announcements (17 male (58.62%), age range 20–39, mean age 25.24 years (SD=6.34)). Adolescents were screened with the structured diagnostic interview “development and well-being assessment” (DAWBA) (Goodman, Ford, Richards, Gatward, & Meltzer, 2000) according to the fourth edition of the diagnostic and statistical manual (DSM-IV) and adults were screened using the “composite international diagnostic interview” (CIDI) (Wittchen & Pfister, 1997). This was done to control for homogeneity among the two groups and to exclude participants with history of psychiatric or neurologic diseases. Adults performed all and exactly the same tasks as adolescents.

All participants were compensated for taking part in this study. All the participants in the adults group, the adolescents and at least one legal guardian for each adolescent gave their written informed consent to participate in the study after receiving a comprehensive description of the study protocol. The study was carried out in accordance with the Declaration of Helsinki. The study was approved by the local research ethics committee.

### 2.2 Apparatus

The stimuli were presented via a head-coil-mounted display system based on LCD technology (NordicNeuroLab AS, Bergen, Norway). Participants responded with a ResponseGrip^TM^ (NordicNeuroLab AS, Bergen, Norway). Stimuli were presented using the Presentation^®^ software (v11.1 Neurobehavioral Systems Inc. Albany CA, USA). Behavioural data was analysed using SPSS (v17.0; LEAD Technologies Inc., Charlotte, NC, USA). Imaging data was analysed using MATLAB (v7.5; MathWorks Company, Natick, MA, USA) and SPM5 (Wellcome Trust, London, UK).

### 2.3 Image acquisition

All MRI data were acquired at the Neuroimaging Centre at the Technische Universität Dresden, using a 3.0 T scanner (Magnetom Tim Trio, Siemens, Erlangen, Germany). Series of T_2_^⁎^-weighted echo-planar images (EPI) with 42 transverse slices tilted approximately 30° towards the coronal beyond the anterior to posterior commissure line, with a 3 mm in-plane resolution and a slice thickness of 2 mm (1 mm gap resulting in a voxel size of 3×3×3 mm^3^), field-of-view (FoV) of 192×192 mm^2^, a flip angle (FA) of 80°, a repetition time (TR) of 2410 ms, a bandwidth of 2112 Hz/pixel, and an echo time (TE) of 25 ms were acquired. The first 3 volumes were discarded to allow the magnetisation to reach equilibrium. High-resolution three-dimensional anatomical images were acquired using a T_1_-weighted magnetisation-prepared, rapid acquisition gradient echo (MP-RAGE) sequence with a FoV=256×224 mm^2^, 176 slices, a voxel size of 1×1×1 mm^3^, a TR of 1900 ms, a TE of 2.26 mm and a FA of 9°.

### 2.4 Task description

**Fig. 2:**
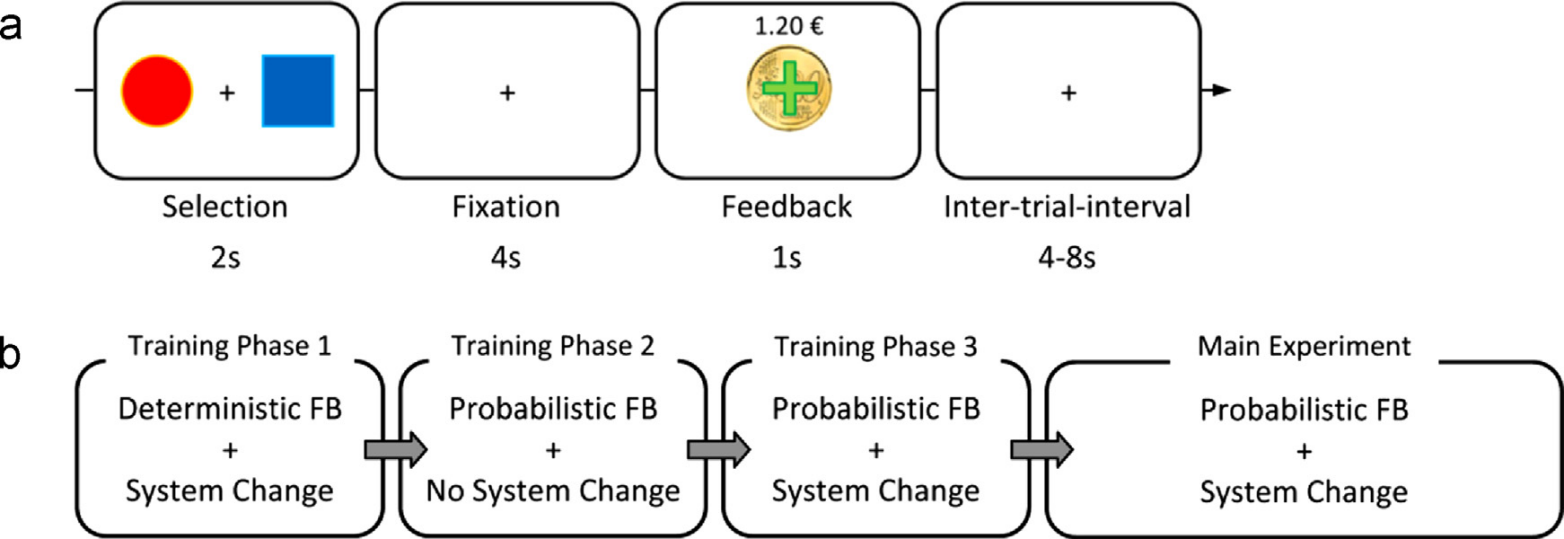
Overview of the experiment (a) A typical trial during the probabilistic reversal learning task and (b) overview of the session. *FB:* Feedback. *System Change* indicates that contingencies were reversed at certain trials.

We employed a probabilistic reversal learning task similar to that used by Hampton et al. (2006). Please see Fig. 2(a) for a detailed description of the procedure of a trial. During each trial, participants were presented with two options, which differed in probabilities of monetary outcomes associated with them. One option was associated with a 70% chance of gaining 20 Euro cents (positive feedback (PFB)) and 30% chance of losing 20 Euro cents (negative feedback (NFB)), while the other option was associated with 40% chance of PFB and 60% chance of NFB. The option with a higher mean payoff (70% PFB: 30% NFB) was designated as the *correct* option, while the other option (40% PFB: 60% NFB) was designated as the *wrong* option. Participants were instructed to maximise their gains, that is, to identify and choose the *correct* option at every trial. At the end of each trial, participants were provided with feedback. Additionally on the feedback screen they were presented with the total amount of money they had accumulated over the preceding trials. Prior to the scan, participants were told that in addition to their participation fee (5 Euros), they would also receive their earnings from the in-scanner task.

Apart from learning to identify the *correct* option during each session, participants also had to adapt to changes in reward contingencies over time. During the session, should the participant choose the *correct* option consecutively four times, contingencies of the options could reverse. That is, the option that was previously *correct* becomes the *wrong* option, and vice versa for the other option. However, as these reversals in contingencies only occurred at a 25% probability after correct responses to at least last four consecutive trials, participants had to remain vigilant in adapting to these changes to maximise their gains. This reward-punishment schedule has been well established in previous probabilistic reversal learning studies (Hampton et al., 2006; Hornak et al., 2004; O׳Doherty, Kringelbach, Rolls, Hornak, & Andrews, 2001).

The in-scanner task consisted of 120 trials. Total task duration was 26 min. Before entering the scanner, participants performed a training session of the task consisting of three phases; see Fig. 2(b). In the first phase of the training session, system changes (change of contingencies) were implemented, but participants were provided with deterministic feedback – that is they were always rewarded for correct responses and punished for wrong responses. The phase ended upon three consecutive system changes. In the second phase, there was no system change, but feedback was probabilistic. The phase ended once the participant has selected the *correct* option consecutively ten times. The third phase combined probabilistic feedback with system changes. This phase was identical to the main task in the scanner. Similar to that of the first phase, this phase ended upon three system changes. Once they have completed their training, the participants proceeded with the in-scanner task.

### 2.5 Behavioural data analysis

Three behavioural performance measures were considered: ratio of correct responses, total accumulated monetary reward and number of system changes. Ratio of correct responses was defined as the ratio of total number of correct responses to total number of trials. On a broad level, the ratio of correct responses reflects how well the participant was able to form associations between the feedback and the options. Number of system changes adds a further dimension as it is dependent on participant׳s understanding of the underlying mechanism of the task, i.e. system changes based on performance. For the purpose of quantifying individual differences in adaptation to a dynamic environment, it is necessary to include both measures to take into account how well they were able to learn the associations and how quickly they were able to adapt to changes.

Ratios of behavioural switch after negative and positive feedbacks, i.e. proportion of behavioural switch after NFB and PFB to the total number of feedbacks were also computed. This parameter was subjected to a mixed-factor analysis of variance (ANOVA) with feedback (NFB/PFB) as within-subject factor and group (adolescents/adults) as between subject factor. Data were checked for normal distribution using a Kolmogorov-Smirnov goodness-of-fit test. The Mann–Whitney U-test was used for non-parametric tests. Mean and standard deviation (SD) values are reported for factors with normal distribution and median for factors with non-normal distribution.

### 2.6 Imaging data analysis

Data was preprocessed to correct for slice timing differences and head motion, spatially normalised to a standard EPI template in MNI space and smoothed with a 8 mm FWHM isotropic Gaussian kernel. Templates were based on the MNI305 stereotaxic space, an approximation of Talairach space (Talairach & Tournoux, 1988).

For the first level analysis, event-related fMRI data were analysed by constructing δ-functions. We constructed a general linear model (GLM) with five regressors: one at the onset of the stimulus, three at the onset of feedback in the current trial and decision in the subsequent trial (PFB, NFB-Stay and NFB-Switch). We did not split trials with PFB into PFB-Stay and PFB-Switch because participants rarely switched their decision after PFB; finally one regressor for trials with no response at the onset of both stimulus and feedback. All of these regressors were convolved with a canonical hemodynamic response function (HRF). In addition, the six scan-to-scan motion parameters produced during spatial realignment were included to account for residual motion effects.

**Fig. 3:**
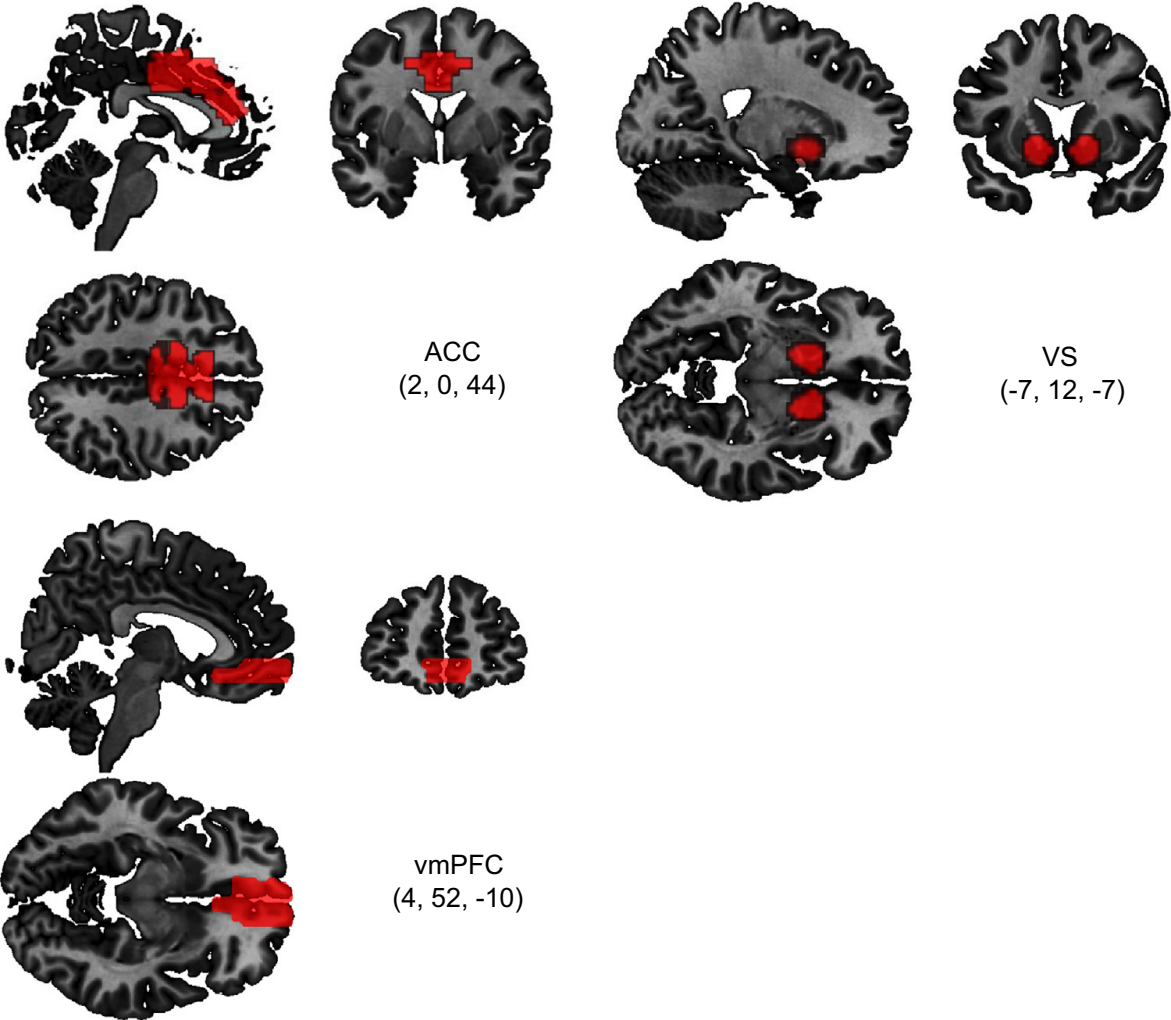
Sections of regions of interest used in the analysis for anterior cingulate cortex (ACC), ventral striatum (VS) and ventromedial prefrontal context (vmPFC). Coordinates shown are in MNI space.

Three regions-of-interest (ROI) were specified: anterior cingulate cortex (ACC), ventromedial prefrontal cortex (vmPFC) and ventral striatum (VS). For the ACC mask, we first combined Brodmann areas 24 and 32 provided in Wake Forest University (WFU) PickAtlas for SPM (Lancaster, Summerln, Rainey, Freitas, & Fox, 1997; Lancaster et al., 2000). It was then masked by a 24 mm radius sphere located at MNI space (0, 27, 45) to remove the pregenual and posterior parts of anterior cingulate. For the vmPFC mask, we used an automated anatomical labelling (AAL) atlas provided in the WFU PickAtlas. For the VS, the regions were specified in accordance to probabilistic maps freely available online (Nielsen & Hansen, 2002). Binary images were made using the threshold value of 0.5 for VS. The selected threshold provided the possibility to cover the whole VS. Finally, the rfxplot toolbox for SPM (Gläscher, 2009) was then used to extract the mean activity elicited by PFB, NFB-Stay and NFB-Switch of the voxels specified by the ROIs. The masks are shown in Figure 3.

Brain imaging data analysis was performed in two steps: (1) the first step used a 2-level randomisation procedure in order to compensate for the imbalance in the number of participants in the groups, non-normal distribution of the data and outliers in the two groups. This procedure was used throughout the imaging data analysis to analyse the main and interaction effects and *post-hoc* tests. (2) In the second step data was categorised into states of activity using the results of the earlier step. This step was used to account for intrinsic differences between adolescents and adults׳ brain activity and base the comparison solely on the significance of differences between the two groups rather their literal magnitudes.

Randomisation procedure, similar to permutation procedure, generates simulated participants based on data acquired from real participants. These generated participants are used to create distributions that are later used for statistical comparison. Participants and distributions are created through *permutation runs*. The first level of randomisation is for generation of simulated participants and the second level of randomisation is for creation of distributions that are subsequently used in statistical test of real data. These randomisation procedures were run on participant and group levels as previously used by Blair and Karniski (1993). On the first level, a randomisation procedure using 200,000×220 permutation runs for the adolescent group and 200,000×28 permutation runs for the adult groups were carried out to generate simulations of participants. In this run, for each simulated participant, three values (for the three conditions of PFB/NFB-Stay/NFB-Switch) were randomly selected (with replacement) from all the values of brain activities, separately for each ROI. Different comparisons were made for the second level of randomisation: main and interaction effects (comparable with main and interaction effects in ANOVA). Subsequently significant and non-significant interactions were subjected to *post-hoc* paired-wise tests with- and with-out groups collapsed, respectively. For this level 500,000 permutation runs were conducted to achieve (i) a distribution of grand difference between adolescents and adults (adolescents vs. adults) in each condition and (ii) distributions of grand differences of mean values over the three conditions (3 comparisons, PFB vs. NFB-Stay, PFB vs. NFB-Switch and NFB-Stay vs. NFB-Switch) for the two groups separately and (iii) the same measures as in (ii) with the two groups pooled together. The second set of distributions are for the case of significant interaction effects and the third set of distributions are for the case of non-significant interaction effects as tested using the first set of distributions. For this level of randomisation, groups of 220 adolescents and groups of 28 adults were randomly selected (with replacement) from the population of simulated participants in each group. These distributions were used to determine the *p*-value of comparison for real data (Hanslmayr, Spitzer, & Bauml, 2009). The distributions of *p*-values for *post-hoc* tests over the three ROIs were corrected for multiple comparisons according to the false discovery rate (FDR) procedure (Benjamini & Hochberg, 1995). We computed a *q* threshold that set the expected rate of false discoveries to 0.029. Furthermore, absolute value of Cliff׳s Delta effect size measure (*δ*) was reported (Cliff, 1993; Macbeth, Razumiejczyk, & Ledesma, 2011). This measure relies on dominance concept rather than mean as in conventional effect size measures such as Cohen׳s *d* and is more robust under skewed distributed data.

In order to compare our 2-level randomisation procedure with standard whole-brain voxel-wise SPM analyses we ran 2×2 full-factorial ANOVAs for each ROI with condition (pairs of three conditions of PFB/NFB-Switch/NFB-Stay) and group as independent factors and percentage signal change as dependent factor. Therefore, similar to the 2-level randomisation procedure we ran 9 (3×3) different 2×2 full-factorial ANOVAs.

The focus of our study was on comparison between adolescents and adults in the pattern of activity of ACC, VS and vmPFC in a probabilistic reversal learning task, i.e. how each group of participants represented feedback and decision in the subsequent trial in these brain areas, rather than comparing brain activities directly between the groups. As such, the BOLD percentage signal change was first converted into *states* of activity, that is, where activity differed significantly between conditions using pair-wise comparisons. Fig. 1 shows an example of this classification. This way, we constructed bi- or tri-state patterns of activity for the three conditions (PFB, NFB-Stay and NFB-Switch) and each brain area.

## 3 Results

### 3.1 Behavioural data

Our results showed that the ratio of correct responses and total accumulated monetary reward were normally distributed, but number of system changes was not. Independent-sample *t*-test on the ratio of correct responses showed no significant difference between groups (adolescents mean (SD)=0.59 (0.07), adults 0.61 (0.06), *t*(246)=1.03, *p*=0.30). Similarly, no significant difference was found for accumulated monetary reward between groups (adolescents 3.58 (1.56), adults 3.51 (1.34), *t*(246)=0.23, *p*=0.81). However, there was significantly higher number of system changes in adults than adolescents (median adolescents 6, adults 7, non-parametric Mann–Whitney *U*-test *Z*=−2.04, *p*=0.04).

A 2×2 mixed-factor ANOVA on ratio of behavioural switch after NFB and PFB revealed significantly higher switching rates in adolescents compared to adults (adolescents=0.28 (0.10), adults 0.23 (0.10), main effect of group: *F*(1, 246)=5.37, *p*=0.02, *η*_*p*_^2^=0.03), significantly higher switching rates after NFB (NFB=0.46 (0.24), PFB=0.06 (0.16), main effect of feedback: *F*(1, 246)=677.15, *p*<0.001, *η*_*p*_^2^=0.73) but a non-significant 2-way interaction (*F*(1, 246)=0.01, *p*=0.92, *η*_*p*_^2^<0.001).

### 3.2 Imaging data

**Fig. 4:**
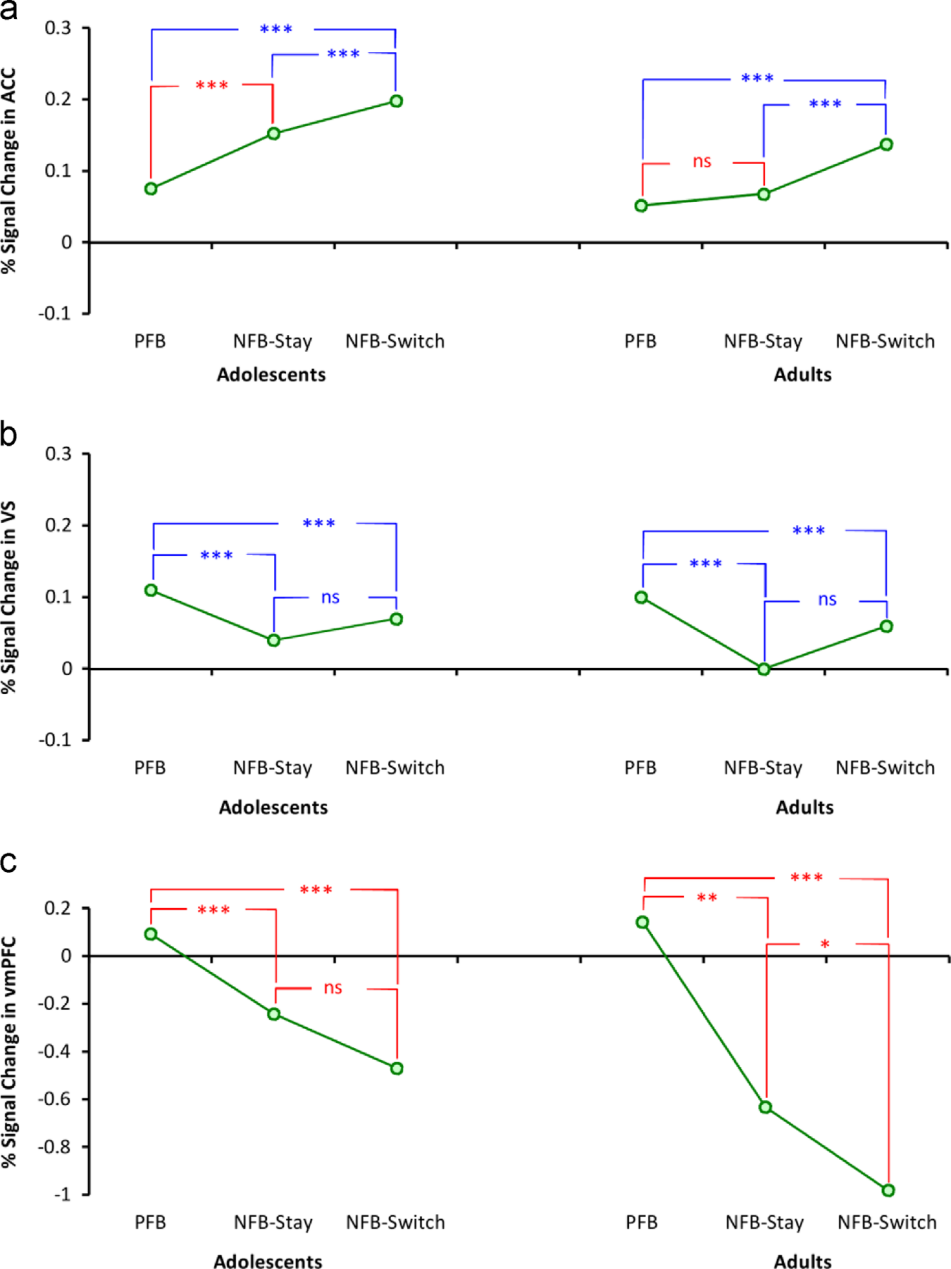
Median of percentage signal change in different regions of interest split over adolescents and adults for the three conditions of positive feedback (PFB), negative feedback stay (NFB-Stay) and negative feedback switch (NFB-Switch). Red marks represent significant interaction and blue marks represent non-significant interaction, see Table 1. For the blue marks the *post-hoc* test was calculated with adolescents and adults groups pooled together. ACC, VS and vmPFC stand for anterior cingulate cortex, ventral striatum and ventromedial prefrontal cortex, respectively. ^⁎^*p*<0.029 (FDR-corrected for multiple comparisons), ^⁎⁎^*p*<0.005, ^⁎⁎⁎^*p*<0.001, and ^ns^ non-significant. (For interpretation of the references to colour in this figure legend, the reader is referred to the web version of this article.)

Our imaging data analysis was focused on the main effects of condition (comparisons of PFB–NFB-Stay, PFB–NFB-Switch, and NFB-Stay–NFB-Switch) and 2-way interaction of group and condition. Table 1 summarise these comparisons and Fig. 4 shows the median of BOLD percentage signal change in different groups and conditions. In order to compare our method with standard whole-brain, voxel-wise SPM analyses we ran 2×2 full-factorial ANOVAs on different ROIs with different condition pairs and groups. Results of these analyses are shown in Table 2. Results showed similar significant differences for the main effect of condition. On the contrary results showed no significant interaction effect. Supplementary material 1 shows box plots of percentage signal change in different conditions.

*Activity in ACC*; PFB elicited a lower BOLD response than NFB (significant main effects of PFB–NFB-Stay and PFB–NFB-Switch). Moreover, there was a stronger BOLD response associated with NFB-Switch than NFB-Stay. The randomisation procedure revealed a significant interaction of group and the condition of PFB–NFB-Stay. *Post-hoc* tests indicated that adolescents showed a significantly higher ACC response to NFB-Stay than PFB, while adults׳ brain activity between NFB-Stay and PFB was similar.

*Activity in* VS; VS activity was substantially higher in PFB than NFB conditions and there was no difference in VS activity between NFB-Stay and NFB-switch conditions. Furthermore, VS activity did not differ between adolescents and adults in any of the comparisons (non-significant interactions).

*Activity in vmPFC*; As in the VS, vmPFC activity was much higher in the PFB condition than in NFB conditions. Moreover, this difference was more pronounced in adults than in adolescents (significant interactions). There was a significant interaction of group and condition in all comparisons. Within-group *post-hoc* tests showed a difference only in the comparison of NFB-Stay and NFB-Switch in which adolescents showed a non-significant difference while adults showed a very highly significant difference. It should be mentioned that, the 2-level randomisation procedure led to 3 significant interactions. The second step of analysis, conversion of continuous values into discrete states of activity, however, revealed a difference in only one of the comparisons (NFB-Stay–NFB-Switch).

To summarise, adolescents and adults differed mainly in ACC activity during the PFB and NFB-Stay conditions and vmPFC activity during the NFB-Stay and NFB-Switch conditions, as marked by ‘†’ in Table 1.

**Fig. 5:**
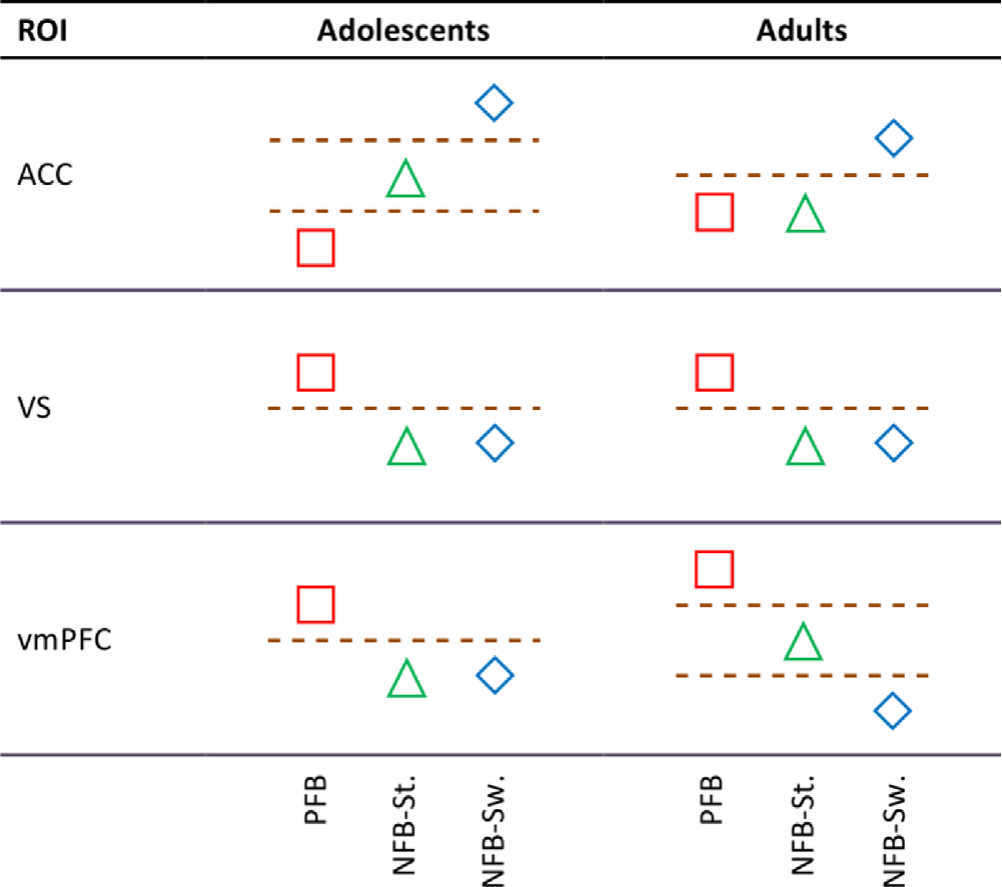
Representation of conditions in ACC, vmPFC and VS in adolescents and adults. Values shown in Fig. 4 are classified into states of activity. Square, triangle and diamond represent the conditions PFB, NFB-Stay and NFB-Switch, respectively.

The continuous BOLD percentage signal change (shown in Fig. 4) was converted into states of activity based on the *p* values of *post-hoc* tests and main effect of condition in the case of significant and non-significant interactions, respectively. The result of this conversion is shown in Fig. 5. Differences between adolescents and adults are reflected in ACC (comparison of PFB and NFB-Stay) and vmPFC (comparison of NFB-Stay and NFB-Switch).

## 4 Discussion

In this study, we tested adolescents and adults to investigate contributions of ACC, VS and vmPFC in feedback-related decision making using a probabilistic reversal learning task. We aimed to investigate how these brain areas represent various conditions of feedback and resulting decision. Subsequent to a 2-level randomisation procedure (1st step) we transformed continuous values of percentage signal change of BOLD signal into discrete states before performing between-group comparisons for different conditions (2nd step). This method revealed differences between the two groups that were not possible to detect using conventional statistical approaches.

Our behavioural data showed a lower number of system changes and a higher ratio of behavioural switch in adolescents compared to adults, but no difference in the ratio of correct responses. Conversion of the brain imaging data into discrete states of activity revealed a difference in the pattern of activity of ACC and vmPFC between adolescents and adults, but no difference in the pattern of activity of VS in response to feedback and subsequent decision (PFB/NFB-Switch/NFB-Stay). These results imply that ACC activity reflected both feedback and decision in adolescents whereas it represented only decision in adults. vmPFC activity represented feedback in adolescents, whereas it reflected both feedback and decision in adults. VS activity reflected solely feedback for both groups.

**Fig. 6:**
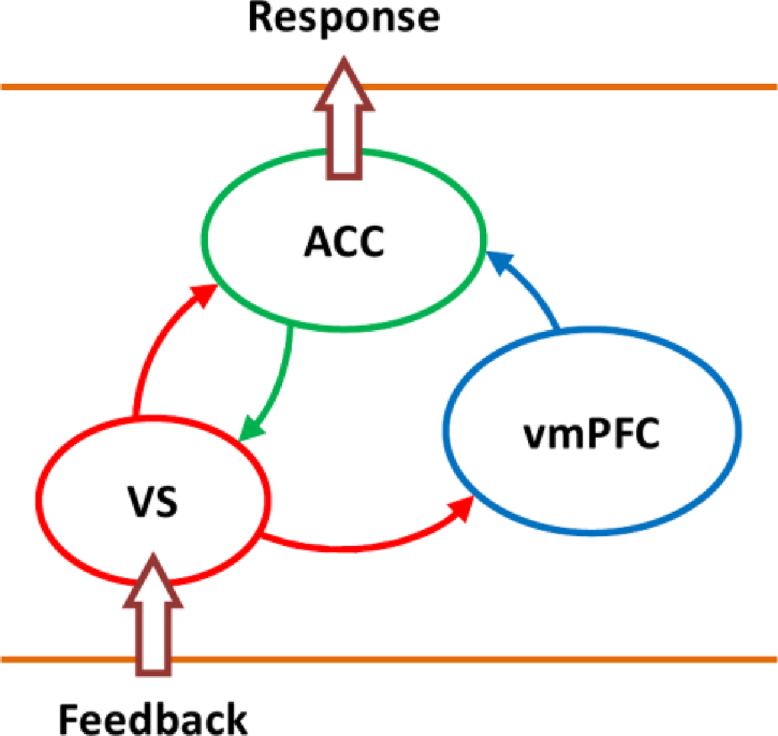
The schematic of the model proposed by Holroyd and Coles (2002), including anterior cingulated cortex (ACC), ventral striatum (VS) and ventromedial prefrontal cortex (vmPFC) in regards to feedback (input) and response (output).

There are several models that explain the decision making network from different perspectives and emphasise different aspects of information processing (Everitt & Robbins, 2005; Frank, 2005) (for reviews see (Grabenhorst & Rolls, 2011; Holroyd & Yeung, 2012; Noonan, Kolling, Walton, & Rushworth, 2012)). Holroyd and Coles (2002) proposed a model which shows the interaction between ACC, VS and vmPFC in decision making, with regard to feedback (input) and decision (output). Fig. 6 shows a schematic of their model. Based on their model, ACC activity reflects the decision based on the provided feedback. This functional role of ACC is also supported by other researchers (Frank, 2005; Frank & Claus, 2006; Shima & Tanji, 1998). According to this model, activity in VS is directly modulated by feedback that additionally incorporates expectation and serves as prediction error, modulated by ACC activity (Behrens, Woolrich, Walton, & Rushworth, 2007). The updated expectation levels are represented in the vmPFC (Grabenhorst & Rolls, 2011) which form the basis of the resultant decision.

ACC, VS and vmPFC activity observed in our adult participants are in line with the model proposed by Holroyd and Coles (2002) with ACC representing decision (Bush et al., 2002; Williams, Bush, Rauch, Cosgrove, & Eskandar, 2004), VS responding to feedback (O׳Doherty, 2004; O׳Doherty, Dayan, Friston, Critchley, & Dolan, 2003; Schlagenhauf et al., 2012; van Leijenhorst et al., 2010), and vmPFC reflecting both feedback and decision (by receiving a modulatory signal from VS) (for a review see (Grabenhorst & Rolls, 2011)). However, our observations in adolescents are not in line with this model as ACC reflected both feedback and decision and vmPFC represented feedback only. We speculate that these observations could be related to poorer feedback-related decision making and probabilistic reversal learning in adolescents.

In two-choice decision tasks, be it deciding between a circle and rectangle or stay and switch, two states of ACC activity is sufficient for the representation of the decision, as shown in adults. This is an efficient way of representation, as the separation of the states is clear. This classification in adolescents, however, is done in three states, which induces complications in interpretation of the activity of ACC in terms of behavioural decision (namely *stay* and *switch*). Having three states of brain activity for two possible options requires finer tuning of threshold of *stay* and *switch* in between PFB and NFB-Stay with NFB-Switch. This also introduces redundant representation of *stay* behavioural decision for PFB and NFB-Stay as both of them lead to *stay*. In other words, it is more accurate (and perhaps easier) for the brain areas responsible to make the final motor action to deal with bi-state representation of decision, as in adults, than tri-state one, as in adolescents.

Another possible drawback of the brain activity in adolescents arises from the activity of vmPFC in response to feedback and subsequent decision. As shown, the activity of vmPFC in adolescents solely represents for feedback which is redundant and identical to the function of the VS. Therefore, its activity does not contribute to the cycle of decision making and expectation update mechanism, which might be a reason for the imprecise representation of decision in ACC.

Our observations suggest that vmPFC could encode an underlying value signal beyond feedback and decision. Adolescents seem to be hypersensitive to NFB, regardless of whether it is relevant (NFB on wrong responses) or misleading NFBs (NFB on correct responses). Further analysis showed that adolescents were more prone to switching behaviour following misleading NFB. That is, they tended to switch more often than adults after receiving misleading NFB (see Supplementary material 2). We speculate that adults were better able to suppress the effects of misleading NFB and stay with their previous decision, while adolescents were more affected by negative feedback. This could reflect a more efficient top-down control (perhaps driven by ACC) in adults while adolescents lacked this cognitive control ability.

To further investigate differential involvement of the ACC and vmPFC between groups in different conditions, we ran a 3-way mixed-factor ANOVA with condition (PFB/NFB-Stay/NFB-Switch), ROI (ACC/vmPFC) as within subject factors and group (adults/adolescents) as between subject factor. This test showed a significant interaction of the three factors *F*(2, 492)=3.234, *p*=0.040, *η*_*p*_^2^=0.013). We should emphasise that the conclusions based on our proposed method of discretisation stand valid without having a significant interaction in an ANOVA. For instance, the outliers in our sample could be arranged in a way that led to a non-significant interaction as the ANOVA does not account for outliers, which are far away from the distribution.

Comparison of our randomisation procedure with standard whole-brain voxel-wise SPM showed similar effects for the main effects. In contrast, this analysis showed no significant interaction. This shows that our approach is not a replication of standard analyses, but reveals effects that are not uncovered by standard analyses. Our novel analysis approach, however, should be validated with conventional analytic approaches in further studies.

Behaviourally, the observations are in line with that of the imaging observations. Adolescents showed a higher rate of behavioural switch and lower number of system changes than adults. Both adolescents and adults had comparable numbers of correct responses. It has to be mentioned that adolescents achieved lower number of system changes. As such, they dealt with a more stable system than adults with significantly higher number of system changes.

To summarise, our results showed a differential representation of feedback and decision in the ACC and vmPFC in adolescents and adults. We argued that this differential representation results in several drawbacks in decision making for adolescents such as redundant and imprecise representation of feedback and decision leading to a higher ratio of behavioural switch and possibly also higher levels of uncertainty. We speculated that adolescents have difficulty in differentially inhibiting negative feedback, reflecting weaker cognitive control. Furthermore, we showed that the functional role of ACC, VS and vmPFC in adults was in-line with the model proposed by Holroyd and Coles (2002) whereas adolescents׳ brain activity in ACC and vmPFC was not in-line with the model.

## Figures and Tables

**Table 1:** Summary of the randomisation procedure comparing the three conditions (PFB/NFB-Stay/NFB-Switch) in adolescents and adults split over the three regions of interest. *Main effect* refers to main effect of condition with groups pooled together. *Interaction* refers to 2-way interaction of condition and group. *Post-hoc* comparisons for different conditions are reported for conditions with significant (*p*<0.05) 2-way interaction of condition and group. ACC, VS and vmPFC stand for anterior cingulate cortex, ventral striatum and ventromedial prefrontal cortex, respectively.

**ROI**	**Condition**	**Main effect**		**Interaction**	***Post-hoc*****test adolescents**		***Post-hoc*****test adults**	
		***p***	***δ***	***p***	***p***	***δ***	***p***	***δ***
ACC	PFB–NFB-Stay	<0.001	0.230	0.015	<0.001†	0.244	0.210†	0.125
	PFB–NFB-Switch	<0.001	0.419	0.057				
	NFB-Stay–NFB-Switch	<0.001	0.192	0.491				
VS	PFB–NFB-Stay	<0.001	0.337	0.162				
	PFB–NFB-Switch	<0.001	0.227	0.369				
	NFB-Stay – NFB-Switch	0.208	0.064	0.284				
vmPFC	PFB–NFB-Stay	<0.001	0.265	0.041	<0.001	0.243	0.003	0.455
	PFB–NFB-Switch	<0.001	0.345	<0.001	<0.001	0.309	<0.001	0.674
	NFB-Stay–NFB-Switch	0.071	0.086	0.010	0.145†	0.068	0.027†	0.275

† Refers to conditions where adolescents and adults differed significantly.

**Table 2:** This table summarises the results of the main effect of condition in 2×2 full-factorial ANOVAs with condition (pairs of three conditions of PFB/NFB-Switch/NFB-Stay) and group as independent factors and percentage signal change as dependent factor with *F*(1, 490). Each coordinate shows the location of peak activity in each specific ROI. Reported *p* values are uncorrected with *k*>10. *ns* stands for non-significant. ACC, VS and vmPFC stand for anterior cingulate cortex, ventral striatum and ventromedial prefrontal cortex, respectively.

**ROI**	**Condition**	***x***	***y***	***z***	***k***	***F***	***p***
ACC	PFB–NFB-Stay	6	24	42	275	81.04	<0.001
		−3	9	51		35.00	<0.001
		9	12	51		27.89	<0.001
	PFB–NFB-Switch	6	24	39	422	61.79	<0.001
		−9	24	33		47.62	<0.001
		−6	9	51		36.47	<0.001
	NFB-Stay–NFB-Switch	9	3	51	306	27.98	<0.001
		−6	3	51		26.63	<0.001
		−9	21	33		24.24	<0.001
VS	PFB–NFB-Stay	12	6	−9	97	102.41	<0.001
		−12	3	−9	67	62.08	<0.001
		−9	18	−12		20.03	<0.001
	PFB–NFB-Switch	9	6	−9	31	34.48	<0.001
		−12	3	−9	19	25.96	<0.001
	NFB-Stay–NFB-Switch						ns
vmPFC	PFB–NFB-Stay	0	54	−3	127	62.79	<0.001
		0	42	−9		59.14	<0.001
		3	30	−12		40.27	<0.001
	PFB–NFB-Switch	−6	36	−9	44	42.02	<0.001
		3	21	−12		21.97	<0.001
	NFB-Stay–NFB-Switch						ns
