# Supplementary material for: Differential representation of feedback and decision in adolescents and adults
Source: Neuropsychologia. 2014 Apr;56(100):280–8. doi: 10.1016/j.neuropsychologia.2014.01.021 (PMC3991323; doi:10.1016/j.neuropsychologia.2014.01.021)
Supplement: Supplementary file 1 — Supplementary data [file mmc1.pdf]

Supplementary Material 1

(a)

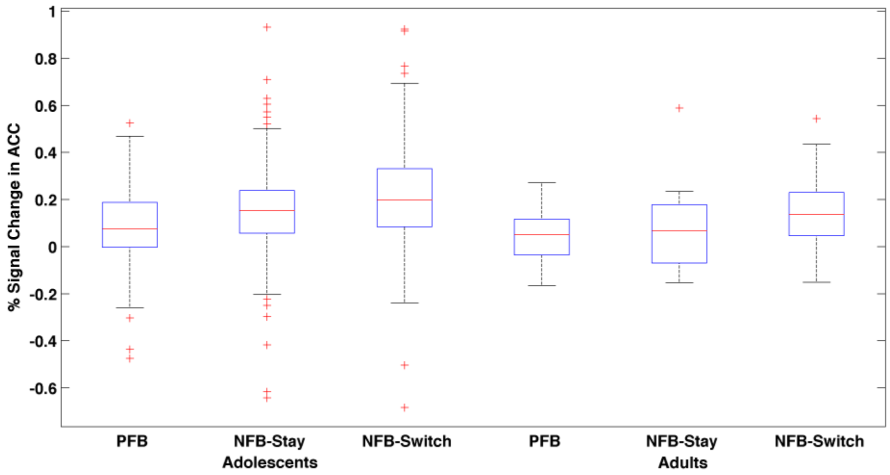

(b)

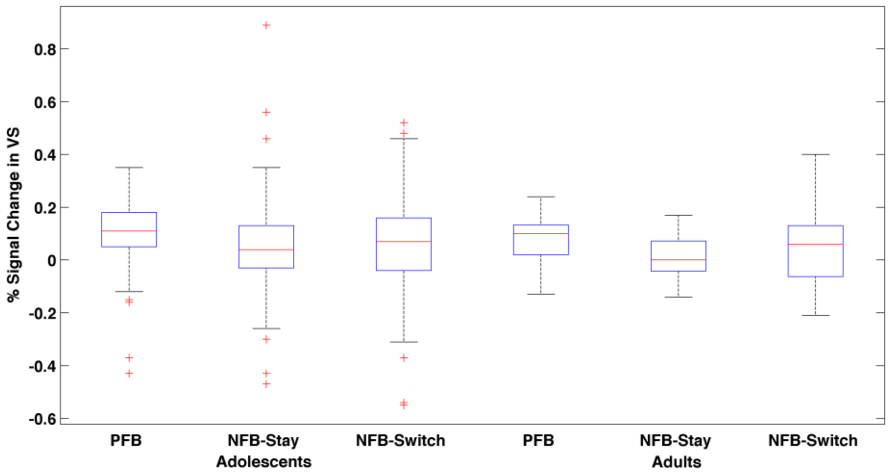

(c)

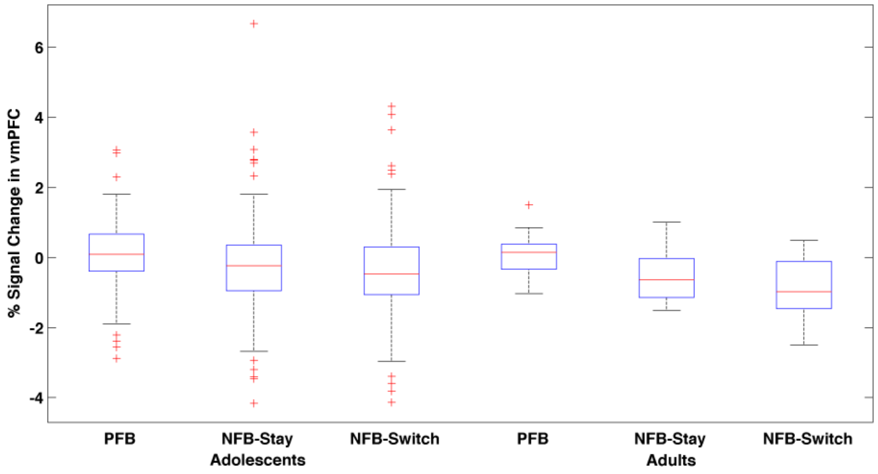

Figure S1. Box plots of percentage signal change in adolescents (the three bars on the left) and adults (the three bars on the right) for three conditions of positive feedback (PFB), negative feed-back-stay (NFB-Stay) and negative feedback-switch (NFB-Switch) for three regions of interest (a) ACC, (b) VS and (c) vmPFC. The central mark is the median, the edges of boxes are the 25th and 75th percentiles, the whiskers extend to the most extreme data points not considered outliers, and outliers are plotted individually.

Supplementary Material 2

We ran a 2 x 2 mixed-factor ANOVA with group as between subject factor and response (correct/wrong) as within subject factor for switching rate after NFB. There was a non-significant main effect of group (adolescents 0.48 (0.15), adults 0.43 (0.15), $F(1, 246) = 2.80$, $p = 0.10$, $\eta_p^2 = 0.01$) but a significant main effect of response (correct 0.39 (0.30), wrong 0.52 (0.22), $F(1, 246) = 77.25$, $p < 0.001$, $\eta_p^2 = 0.24$) and a significant 2-way interaction of group and response ($F(1, 246) = 3.90$, $p = 0 .05$, $\eta_p^2 = 0.02$). Subsequently, we ran independent-sample *t*-tests for correct and wrong responses to compare adolescents' and adults' switching rate after NFB. The results showed that adolescents switched significantly more often on correct responses after NFB (adolescents 0.43 (0.19), adults 0.35 (0.20), $t(246) = 2.10$, $p = 0.04$) but there was no significant difference on wrong responses after NFB (adolescents 0.53 (0.15), adults 0.51 (0.13), $t(246) = 0.75$, $p = 0.45$).
